# A Case of Appendicitis Due to Burkitt Lymphoma Masking the Systemic Symptoms of Rapidly Progressing Burkitt Lymphoma

**DOI:** 10.70352/scrj.cr.24-0178

**Published:** 2025-02-28

**Authors:** Tomoya Masuda, Ryoma Sugimoto, Kenta Kobashi, Hiroshi Ishii, Kensuke Tsunemitsu

**Affiliations:** Department of Surgery, Saiseikai Saijo Hospital, Saijo, Ehime, Japan

**Keywords:** Burkitt lymphoma, appendiceal tumor, appendicitis

## Abstract

**INTRODUCTION:**

Primary malignant lymphoma of the appendix is a rare disease, and primary Burkitt lymphoma of the appendix has been reported very rarely in Japan. Burkitt lymphoma is an aggressive lymphoma that progresses more rapidly than other malignant lymphomas, making it sometimes difficult to distinguish between systemic symptoms, such as fever associated with lymphoma progression and fever caused by appendicitis.

**CASE PRESENTATION:**

A 21-year-old man underwent open appendectomy after antibiotic treatment for acute appendicitis proved ineffective. Postoperative pathological findings confirmed acute appendicitis. Antibiotics were continued after surgery, and the patient’s fever and abdominal symptoms gradually improved. However, abdominal distension recurred on the 18th day of hospitalization. Blood tests showed a re-elevation of the white blood cell count, suggesting a postoperative intraperitoneal abscess. Despite further antibiotic treatment, fever and leukocytosis persisted. On the 28th day of hospitalization, abnormal lymphocytes were detected in the peripheral blood, and we realized that the persistent fever was due to systemic symptoms of malignant lymphoma rather than a complication of appendicitis. On the 30th day, the patient was referred to the hematology department and subsequently diagnosed with Burkitt lymphoma. Chemotherapy was initiated on the 40th day of hospitalization. At the time of this writing, the patient had remained alive without recurrence for 4 years 3 months postoperatively.

**CONCLUSION:**

In this case, symptoms of acute appendicitis and systemic symptoms of malignant lymphoma appeared simultaneously. As a result, the systemic symptoms of malignant lymphoma were misdiagnosed as postoperative complications, leading to a delay in diagnosis. Primary appendiceal Burkitt lymphoma is extremely rare, and its clinical features remain unknown. It is important to recognize that primary appendiceal Burkitt lymphoma can present with systemic symptoms concurrently with appendicitis. Surgeons should be aware of the clinical features of appendicitis caused by Burkitt lymphoma, which differ from those caused by other appendiceal tumors or malignant lymphoma.

## Abbreviations

LDH
: lactate dehydrogenase
CT
: computed tomography
CVAD/MA
: cyclophosphamide, vincristine, doxorubicin, dexamethasone, methotrexate, and cytarabine

## INTRODUCTION

Primary malignant lymphoma of the appendix is a rare disease,^1)^ particularly primary Burkitt lymphoma of the appendix.^2)^ Burkitt lymphoma is an aggressive lymphoma that progresses more rapidly than other malignant lymphomas.^3,4)^ Therefore, it can sometimes be difficult to distinguish between systemic symptoms, such as fever associated with lymphoma progression, and fever related to appendicitis and its complications.

## CASE PRESENTATION

A 21-year-old man presented with no notable family or medical history. He had been constipated for approximately 1 week and was rushed to our hospital with the complaint of right lower abdominal pain. On admission, his temperature was 36.5°C, blood pressure was 114/86 mmHg, and pulse rate was 101 beats/min. There was localized pressure and peritoneal irritation in the right lower abdomen, with the strongest point of tenderness also in this region.

**Fig. 1:**
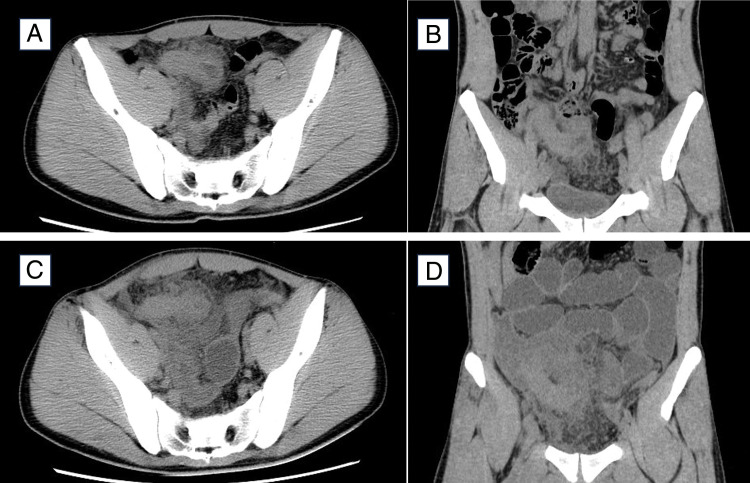
(**A, B**) On the first day, the appendix was swollen to 30 mm, and surrounding panniculitis was evident. (**C, D**) On the fourth day, the appendiceal swelling worsened, and ileal dilatation and fluid retention were observed.

Blood tests revealed a white blood cell count of 16800/μL and a neutrophil count of 84.3%. The C-reactive protein level was 8.85 mg/dL, and the lactate dehydrogenase (LDH) level was 122 IU/L (reference range: 124–222 IU/L). Computed tomography (CT) showed that the appendix was swollen to 30 mm with surrounding panniculitis (**Figs. 1A** and **1B**). No swollen lymph nodes were observed on CT. The patient was diagnosed with acute appendicitis, and the antibiotic cefmetazole was administered.

From the second day of hospitalization, the patient continued to have a fever of 39°C, with no improvement in abdominal symptoms or blood test results. A repeat CT examination on the fourth day showed worsening appendiceal swelling, ileal dilatation, and fluid retention (**Figs. 1C** and **1D**). The patient was diagnosed with paralytic ileus due to worsening appendicitis. Antibiotics were judged ineffective, and surgery was performed. Open appendectomy via a midline lower abdominal incision was chosen because laparoscopic surgery was considered difficult due to the ileus. The case appeared to be a typical appendicitis with abscess formation. No other tumor lesions, including swollen lymph nodes, were identified intraoperatively.

**Fig. 2:**
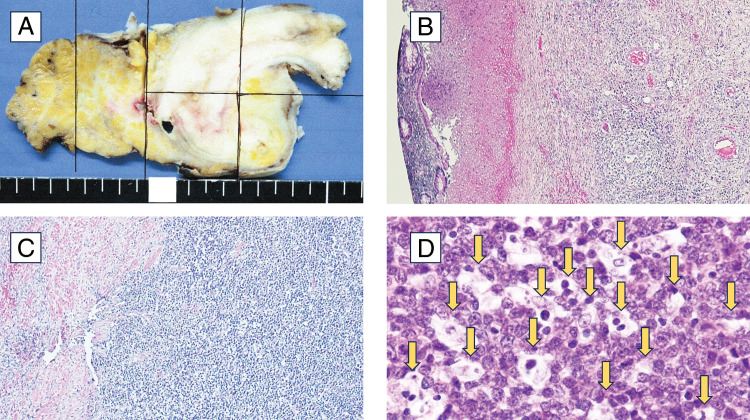
(**A**) Post-fixation appendix: Macroscopically, no differences from normal appendicitis were observed (black line: pathological section line). (**B**) Low magnification: Falling off of the appendiceal mucosa and neutrophil infiltration from the mucosa to the subserosa were observed. (**C**) Low magnification: Numerous lymphocytic infiltrations were observed from the mucosa to the subserosa, suggesting the possibility of a concomitant lymphoproliferative disease. (**D**) High magnification: The starry sky appearance, characteristic of this disease, was observed.

Pathological examination of the resected appendix revealed mucosal shedding and neutrophil infiltration extending from the mucosa to the subserosa, consistent with acute appendicitis (**Figs. 2A** and **2B**). However, numerous lymphocytic infiltrations from the mucosa to the subserosa raised the possibility of a concomitant lymphoproliferative disease (**Fig. 2C**).

**Fig. 3:**
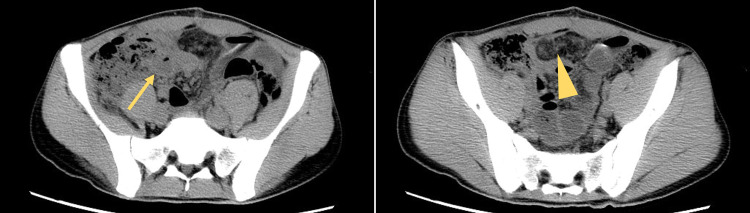
Computed tomography revealed ascites (arrow) and panniculitis (arrowhead) in the pelvic cavity, and an abscess was suspected.

Postoperatively, the patient continued to have a fever of 37°C to 38°C from the 5th to 10th day of hospitalization, and antibiotics were continued. Cefmetazole was replaced with piperacillin–tazobactam, after which the symptoms improved. However, the patient developed abdominal distension on the 18th day, and blood tests showed a re-elevation of the white blood cell count (13700/μL). CT revealed ascites (arrow) and pelvic panniculitis (arrowhead), suggesting an abscess (**Fig. 3**). Antibiotic therapy was resumed from the 18th to 23rd day of hospitalization, and cefmetazole was reintroduced because of prolonged tazobactam administration.

Although the C-reactive protein level remained low postoperatively, the patient continued to have a fever (37°C–38°C) and a persistently elevated white blood cell count (9100–10100/μL). On the 28th day, the white blood cell count re-elevated to 21800/μL, and abnormal lymphocytes were detected. The LDH level was elevated at 458 IU/L, and the interleukin-2 receptor level was 685 U/mL (reference range: 157–474 U/mL). Given the appendiceal findings suggestive of malignant lymphoma, the persistent fever and leukocytosis were suspected to have resulted from systemic symptoms of lymphoma rather than complications of appendicitis.

On the 30th day, a hematology consultation led to a diagnosis of malignant lymphoma. Immunohistochemistry revealed CD3(−), CD5(−), CD10(−), CD20(+), CD79a(+), Bcl-2(−), cyclin D1(−), and Epstein-Barr virus-encoded small RNAs(−). Hematoxylin–eosin staining showed the characteristic “starry sky” appearance (**Fig. 2D**). Fluorescence in situ hybridization confirmed a c-Myc translocation, leading to a diagnosis of Burkitt lymphoma.

**Fig. 4:**
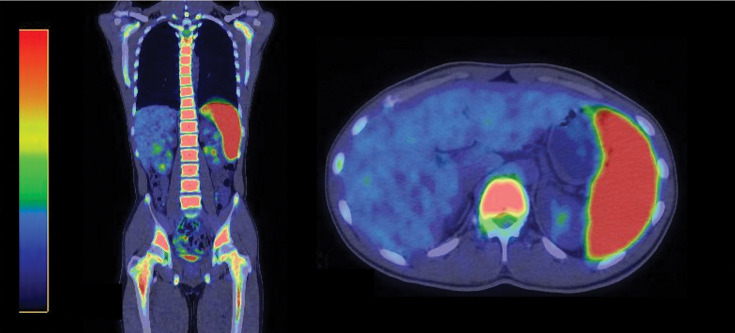
Positron emission tomography–computed tomography showed diffuse abnormal accumulation of fluorodeoxyglucose in the bone marrow. The splenomegaly rapidly worsened within 1 month, and abnormal fluorodeoxyglucose accumulation was observed in the spleen.

Positron emission tomography–CT on the 32nd day showed diffuse abnormal fluorodeoxyglucose accumulation in the bone marrow. The patient was diagnosed with stage IV Burkitt lymphoma. The abnormal lymphocytes in the peripheral blood were thought to be due to bone marrow infiltration. Splenomegaly progressed rapidly over 1 month, and abnormal fluorodeoxyglucose accumulation was also observed in the spleen (**Fig. 4**). No other abnormal accumulations suggestive of tumor lesions were found.

**Fig. 5:**
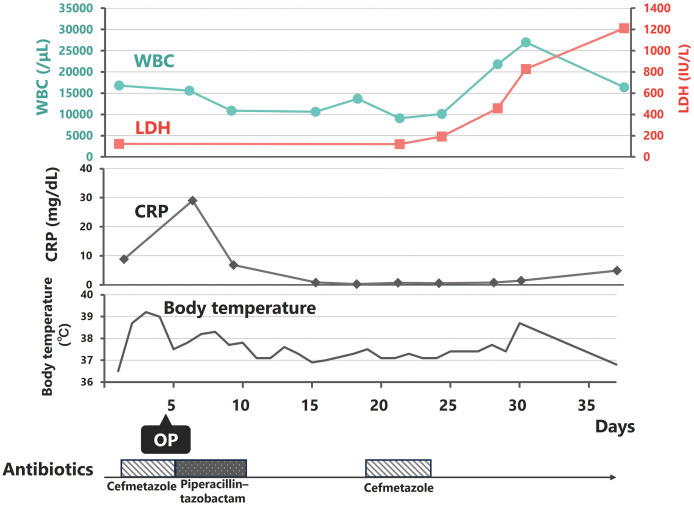
Clinical course. WBC, white blood cell; LDH, lactate dehydrogenase; CRP, C-reactive protein

Chemotherapy (hyper-CVAD/MA regimen: cyclophosphamide, vincristine, doxorubicin, dexamethasone, methotrexate, and cytarabine) was initiated on the 40th day of hospitalization. The clinical course is shown in **Fig. 5**. The patient achieved complete remission. At the time of this writing, he had remained alive without recurrence for 4 years and 3 months postoperatively.

## DISCUSSION

This case highlights that primary appendiceal Burkitt lymphoma is an extremely rare disease, and its clinical features remain largely unknown. In this case, symptoms of acute appendicitis and systemic symptoms of malignant lymphoma appeared simultaneously. As a result, the systemic symptoms of malignant lymphoma were initially misdiagnosed as postoperative complications, leading to a delay in the diagnosis and initiation of treatment.

In the United States, primary appendiceal malignant tumors account for only 0.7% of primary intestinal malignant tumors. The most common types are adenocarcinoma (65.4%), neuroendocrine tumors (31.7%), and malignant lymphoma (1.7%), with malignant lymphoma being rare among primary appendiceal tumors.^1)^ It has been reported that diffuse large B-cell lymphoma (34.5%) is the most common type of primary appendiceal malignant lymphoma, followed by Burkitt lymphoma (25.9%). However, primary appendiceal malignant lymphoma remains a rare disease, and the number of reported cases is very small.^2)^ In Japan, primary appendiceal Burkitt lymphoma is extremely rare, with only 3 reported cases, and the incidence is believed to be even lower than in the United States.^5–7)^ None of these reports mentioned the simultaneous appearance of appendicitis and systemic symptoms. When primary Burkitt lymphoma of the appendix causes appendicitis, the physical findings and symptoms are not significantly different from those of appendicitis of other causes. However, appendicitis caused by Burkitt lymphoma is distinct in that its symptoms overlap with lymphoma-associated systemic symptoms, such as fever. Surgery is essential for a definitive diagnosis because preoperative diagnosis is extremely challenging. Notably, it has been reported that in 86% of cases of tumor-induced appendicitis, the appendiceal diameter on CT is ≥15mm.^8)^ In this case, we should have suspected that the tumor was the cause of the appendicitis prior to surgery. While appendicitis is now often treated conservatively with antibiotics, signs suggestive of an appendiceal tumor—such as a large appendiceal diameter—underscore the importance of early surgery and diagnosis.

Burkitt lymphoma is a highly aggressive, rapidly growing B-cell non-Hodgkin lymphoma that requires prompt diagnosis and initiation of treatment.^3,4)^ In this case, the patient continued to have a fever of >37°C and an elevated white blood cell count even after appendectomy. Initially, we suspected an intraperitoneal abscess as a postoperative complication of appendicitis. However, with the sharp rise in LDH levels 2 weeks after surgery, we concluded that the Burkitt lymphoma had rapidly worsened over a short period, resulting in the onset of systemic symptoms. On the 10th day of hospitalization, pathological findings revealed lymphocytic proliferation, raising the possibility of primary appendiceal malignant lymphoma. However, we did not attribute the nonspecific postoperative course to malignant lymphoma until abnormal lymphocytes were detected in the peripheral blood on the 28th day of hospitalization.

Acute appendicitis due to luminal obstruction is the most common presentation for most tumor types.^9)^ However, tumors localized to the appendix rarely cause symptoms other than appendicitis. Unlike primary appendiceal adenocarcinoma and primary appendiceal neuroendocrine tumors, primary appendiceal malignant lymphoma can present with systemic symptoms. Burkitt lymphoma, in particular, tends to cause extranodal infiltration of the bone marrow and central nervous system.^3,10)^ In this case, no other lesions were found in the abdominal cavity, but the lymphoma had infiltrated the bone marrow by the 32nd day of hospitalization. When surgeons focus solely on postoperative care following appendicitis, there is a risk of overlooking the progression of malignant lymphoma. In fact, in this case, bone marrow infiltration was not detected until a positron emission tomography–CT scan was performed.

While Burkitt lymphoma is highly malignant, it is also highly sensitive to chemotherapy. The overall survival rate in adult cases has been reported to be 75%–85%.^3)^ Early diagnosis and prompt treatment are crucial for improving prognosis. In this case, although it took 30 days from the initial consultation to diagnosis, treatment was initiated on the 40th day of hospitalization, and the patient had a favorable outcome. However, treatment for malignant lymphoma was delayed because we initially assumed the symptoms were complications of appendicitis. This is an important point of reflection for clinicians.

In previously reported cases of primary appendiceal Burkitt lymphoma, the diagnosis was often made early based on postoperative pathological findings. However, in some cases, including the present case, the diagnosis was delayed because the postoperative course was atypical.^6,11)^ In these reports, the pathological diagnosis of acute appendicitis initially masked the systemic symptoms caused by malignant lymphoma. Burkitt lymphoma progresses extremely rapidly and is prone to causing systemic symptoms concurrently with appendicitis. Therefore, it is crucial to pay close attention to systemic symptoms, even more so than with other malignant lymphomas.

## CONCLUSION

We encountered a case of appendicitis caused by Burkitt lymphoma, which masked the systemic symptoms of rapidly progressing Burkitt lymphoma. Primary appendiceal Burkitt lymphoma is extremely rare, and its clinical features remain largely unknown. It is important to recognize that primary appendiceal Burkitt lymphoma can present with systemic symptoms concurrently with appendicitis. Awareness of the clinical features of primary appendiceal Burkitt lymphoma can help clinicians optimize treatment and outcomes.

## ACKNOWLEDGMENTS

We would like to thank Haruhiko Otani from the Radiology Department and Norifumi Ueda from the Pathology Department of our hospital for their helpful discussions and comments. We also thank Angela Morben, DVM, ELS, from Edanz (https://jp.edanz.com/ac) for editing a draft of this manuscript.

## DECLARATIONS

### Funding

None of the authors received any funding for this study.

### Authors’ contributions

TM wrote the manuscript.

All authors read and approved the final manuscript.

### Availability of data and materials

Not applicable.

### Ethics approval and consent to participate

Not applicable/This work does not require ethical considerations or approval.

### Consent for publication

Written informed consent for publication was obtained from the patient.

### Competing interests

The authors declare that they have no competing interests.
